# Management of chronic Achilles ruptures: a scoping review

**DOI:** 10.1007/s00264-021-05102-5

**Published:** 2021-06-05

**Authors:** Zaki Arshad, Edward Jun Shing Lau, Shu Hui Leow, Maneesh Bhatia

**Affiliations:** 1 School of Clinical Medicine, University of Cambridge, Cambridge Biomedical Campus, Box 111, Cambridge, CB2 0SP UK; 2 Department of Trauma and Orthopaedic Surgery, University Hospitals Leicester NHS Trust, University Hospitals of Leicester Headquarters, Balmoral Building, Level 3, Leicester, UK

**Keywords:** Achilles, Chronic rupture, Achilles tendon rupture, Scoping review, Neglected rupture

## Abstract

**Purpose:**

This scoping review aims to systematically map and summarise the available evidence on the management of chronic Achilles ruptures, whilst identifying prognostic factors and areas of future research.

**Methods:**

A scoping review was performed according to the frameworks of Arksey and O’Malley, Levac and Peters. A computer-based search was performed in PubMed, Embase, EmCare, CINAHL, ISI Web of Science and Scopus, for articles reporting treatment of chronic Achilles ruptures. Two reviewers independently performed title/abstract and full text screening according to pre-defined selection criteria.

**Results:**

A total of 747 unique articles were identified, of which 73 (9.8%) met all inclusion criteria. A variety of methods are described, with flexor hallucis longus tendon transfer being the most common. The most commonly reported outcome is the American Orthopaedic Foot and Ankle Society (AOFAS) score, although 16 other measures were reported in the literatures. All studies comparing pre- and post-operative outcomes reported significant post-treatment improvement. Complications were reported in 50 studies, with an overall pooled complication rate of 168/1065 (15.8%).

**Conclusion:**

Although beneficial results were reported following a variety of techniques, comparison between these is challenging due to the low-level study designs used and confounding factors such as treatment delay and tendon gap size. Further research comparing the efficacy of different techniques is required in order to facilitate the development of an evidence-based treatment protocol. Such work would allow clinicians to better understand the suitability of the large variety of reported techniques and select the optimal strategy for each individual patient.

**Supplementary Information:**

The online version contains supplementary material available at 10.1007/s00264-021-05102-5.

## Introduction

Rupture of the Achilles tendon is a relatively common injury, with around 4500 Achilles ruptures occurring in the UK every year. Recent epidemiological data demonstrates a significant 39% rise in incidence, from 1.8 per 100,000 person years in the USA in 2012 to 2.5 per 100,000 person years in 2016. A similar trend is also reported in a number of other countries [1–4]. Given that the majority of Achilles ruptures occur during participation in sports such as basketball, numerous authors suggest that this increasing incidence may be due to an increase in participation in recreation sports, particularly in older adults. Other potential factors include an increased awareness and therefore diagnosis of ruptures by emergency doctors, although there is currently no strong evidence to support either hypothesis.

Treatment of acute ruptures is widely debated with previous research describing both operative and conservative (functional dynamic regime) methods [5–7]. Traditionally, open operative repair has been the favoured option with authors showing lower re-rupture rates compared to nonoperative methods [8, 9]. More recently, however, a number of authors have reported excellent outcomes and lower re-rupture rates, with the use of nonoperative functional orthotic treatment, such as the Leicester Achilles Management Protocol (LAMP) and Swansea Morriston Achilles Rupture Treatment (SMART) protocol. Research also suggests that nonoperative management is associated with fewer short-term complications. The emergence of this new evidence has led to non-operative treatment becoming the mainstay of contemporary treatment protocols. If the initial tendon rupture is not diagnosed promptly, as is the case in up to 20% of patients, the injury may then be termed chronic or neglected [10]. Authors disagree as to the exact definition of a chronic lesion; however, a recent systematic review by Flint et al. suggests that the term chronic should be used to define a rupture presenting at least four weeks after the initial injury [11].

A wide variety of techniques such as flexor hallucis longus tendon transfer and V–Y plasty [12–14] have been described in the management of chronic Achilles ruptures. To the best of our knowledge, the only previous scoping/systematic review investigating the full breadth of treatment options was published in 2013, with 34 studies included. However, since that date, there has been a surge in publications reporting treatment of chronic Achilles ruptures, using various techniques. There is therefore a gap in the current literature for an up-to-date review of management techniques. This scoping review addresses this by systematically mapping and summarising current evidence regarding the management of chronic Achilles ruptures, whilst identifying areas for future research. This aims to improve readers’ knowledge of the available treatment strategies and associated outcomes, and aid clinicians in optimising treatment protocols.

## Methods

A scoping review methodology was chosen for this article due to the broad aim of systematically mapping and summarising the full breadth of literature regarding the treatment of chronic Achilles ruptures. Methodological guidelines for the conductance of scoping reviews have been developed by Arksey and O’Malley, Levac and The Joanna Briggs Institute [15–17]. This review adheres to these guidelines, which all describe five key stages in the conductance of a scoping review, as detailed below.

### Identifying the research question

The following research questions were developed to guide this review:

- What management options are currently reported for the management of chronic Achilles ruptures and what are their outcomes?
- If possible to compare outcomes, which techniques have the greatest efficacy?
- What prognostic factors may influence treatment outcome?

### Identification of relevant studies

A thorough computer-based search was performed in six electronic databases including: PubMed, Embase, EmCare, CINAHL, ISI Web of Science and Scopus. A combination of free text and medical subject heading (MeSH) terms such as ‘Achilles’, ‘tendoachill*’, ‘calcaneal tendon’, ‘rupture’, ‘chronic’ and ‘neglected’ was used (see online resource 1 for full details). The Boolean operators ‘and’ and ‘or’ were used to combine terms in full search strings. All searches were performed with an English language restriction (as the research team lacks translation capabilities) and no date restrictions. Searches were conducted on 15 February 2021. Manual reference list analysis of review articles was performed to ensure retrieval of all relevant articles.

### Study selection

Following article retrieval, all studies were imported into Rayyan systematic reviews web application to aid the screening process [18]. Two authors performed two-stage screening, initially involving title/abstract screening and then full text screening, guided by the selection criteria below:

1. *Population:* Patients of all ages with chronic Achilles ruptures. Due to the large variation in the time period used to define a chronic rupture, no restriction as to the minimum duration between injury and diagnosis/treatment was imposed. All studies describing treatment of ‘chronic’ or ‘neglected’ Achilles ruptures were included.
2. *Intervention:* Any intervention for the management of chronic Achilles rupture.
3. *Comparison:* A comparison group was not required for inclusion in this review.
4. *Outcomes:* Studies reporting outcomes using any validated or non-validated scores were included. Examples of scores include American Foot and Ankle Society (AOFAS) score, Achilles Tendon Total Rupture Score (ATRS), Leppilahti score, Tegner Score, Hooker scale and 36-item short-form survey (SF-36). Studies reporting outcomes only in terms of patient-reported satisfaction or symptom improvement were excluded.
5. *Study design:* Original research studies (observational studies, cohort studies, randomised controlled trials) were included. Review articles, case reports, commentaries and abstracts were excluded. Studies failing to report treatment outcomes for Achilles rupture separately from other conditions, for example, Achilles tendinosis, were excluded.
6. *Date:* No publication date inclusion criteria were imposed at either the search or screening stage.
7. *Language:* Studies published in the English language were included. Due to the lack of funding and linguistic capabilities of the research team, studies published in all other languages were excluded.

### Charting the data

A pilot data extraction form was created following discussion between the review team. This sheet contained the following headings:

- Author
- Year of publication
- Type of study
- Number of patients
- Mean age
- Male: female ratio
- Treatment method
- Mean size of tendon defect
- Mean treatment delay
- Outcome scores, e.g. AOFAS, SF-36, ATRS, etc.
- Significant difference between pre- and post-operative score
- Any comparison group and outcome comparison?
- Re-rupture rate
- Complications
- Follow-up period

Two reviewers independently used this form to extract data from the first ten relevant studies. Discussion then took place as to the suitability of the form [19], at which point the decision was taken to add two further headings, ‘minimum treatment delay for inclusion’ and ‘prognostic factors’. Once these headings were added, the final sheet was used to extract relevant data from all studies.

### Collating, summarising and reporting the results

Study results are reported in a qualitative thematic manner, with distinct sections focussing on key themes such as the outcome measures used, treatment results and complications. Basic study characteristics including year of publication, number of patients, mean age, male:female ratio and follow-up period are displayed in Table 1. The number of studies retrieved using the search strategy and excluded at both the title/abstract and full text screening stage is detailed in a PRISMA flow diagram [20]. Outcome scores were pooled across studies reporting the same treatment technique if there were at least five studies reporting a particular technique and if at least three of these studies reported both pre- and post-operative outcome scores. Pooling was performed in R 4.0.0 software (R foundation for statistical computing, Vienna, Austria), using DerSimonian and Laird random effects weighting. Missing standard deviation values were imputed according to the method of Walter and Yao [21]. The pooled pre-operative outcome score was subtracted from the equivalent pooled post-operative score to calculate an unstandardised mean difference. Studies which did not record both a pre- and post-operative score were not included in this analysis.

**Table 1:** Summary study characteristics including first author, year of publication, type of study, number of patients, male:female sex ratio (M:F), mean age, mean delay between injury and treatment and mean follow-up period

Author	Year	Type of study	Number of patients	M:F	Mean age in years (range)	Mean delay in weeks	Mean follow-up in months
Abubeih [22]	2018	Case series	21	15:6	40.2 (16–70)	8.8 (5–18)	15 (12–24)
Ahmad [23]	2016	Case series	32	20:12	53.3 (20–74)	14.6 (4.3–45)	62.3 (18–150)
Alhaug [24]	2019	Case series	21	15:6	54.5 (32–77)	NA	49
Arthur [25]	2020	Case series	7	NA	NA	30 (9–96)	38 (17–67)
Badalihan [26]	2015	Case series	51	42:9	38.4 (20–48)	18.1 (8–24)	24 (1.2–57.6)
Bai [27]	2019	Cohort	26	25:1	36.7 (22–53)	NA	19.5 (24–42)
Baumfield [28]	2017	Case series	6	4:2	50 (33–65)	6–36	9 (5–12)
Becher [29]	2018	Case series	14	12:2	57 (40–71)	> 4	67.2 ± 19.2
Bertelli [30]	2009	Case series	20	18:2	74 (65–82)	14 (7–23)	Minimum 12
Borah [31]	2020	Case series	5	3:2	30–55	6–10	12
Coull [32]	2003	Case series	16	NA	32–79	> 4	5–120
Elgohary [33]	2016	Case series	19	13:6	46 (24–62)	16 (8–26)	29 (13–52)
El Shazly [34]	2014	Case series	15	12:3	37.7 (27–51)	13.5 (7–26)	27 (24–33)
El-Shewy [35]	2009	Case series	11	9:2	34.3 (23–29.5)	15 (11–23)	(72 -108)
Elias [36]	2007	Case series	15	10:5	55.8 (39–74)	17.3 (1–57)	4 (0.3–13.3)
Esenyel [37]	2014	Case series	10	10:0	41 (38–45)	8.3 (4.3–13.0)	43.2 (24–60)
Fotiadis [38]	2007	Case series	9	8:1	41 (35–46)	NA	43.9 (24–72)
Gedam [39]	2016	Case series	14	11:3	45.6 (27–63)	23.6 (8.6 – 42.6)	30.1 (12–78)
Guclu [40]	2016	Case series	17	12:5	33 ± 7	30 (17.2–51.4)	195 (158–226)
Hahn [41]	2008	Case series	7	4:3	36–72	17.4–417.1	29–62
Hollawell [42]	2015	Case series	4	4:0	50 (40–63)	11.5 (8–16)	37.3 (15.3–51.5)
Ibrahim [10]	2009	Case series	14	14:0	41.6 ± 3.1	15 ± 15	28–41
Ibrahim [43]	2007	Case series	13	13:0	43 (29–50)	15 (10–43)	45
Jain [44]	2020	Case series	15	9:6	43.5 ± 12.4	NA	19.1 (13–24)
Jennings [45]	2002	Case series	16	6:10	52 (27–78)	NA	36 (6–96)
Jiang [46]	2019	Case series	7	6:1	47.3 (37–56)	> 6	31.3 (26–36)
Jielile [47]	2016	Cohort	57	48:9	36.5 (29–47)	NA	24 months
Khalid [48]	2018	Case series	10	5:5	58.4	NA	30.9 (17–43)
Khiami [49]	2013	Case series	23	20:3	52.1 (28–79)	57.4 (12.6–123.4)	24.5 (12–43)
Koh [50]	2019	Cohort	49	26:23	58.4	17.6	12
Kosaka [51]	2011	Case series	20	14:6	43 (22–65)	NA	164 (124–224)
Kosanovic [52]	2008	Case series	22	20:2	50 (29–72)	7.1 (4–40)	67 (14–176)
Lee [53]	2007	Case series	9	6:3	58.2 (25–85)	94.3 (38.6 – 257.1)	20–30
Lin [13]	2016	Case series	29	23:6	40.3 (19.2–71.5)	NA	31 (13–68)
Lin [54]	2019	Case series	20	16:4	38 (20–71)	20.4 (4–96)	32.8 (12–68)
Lins [55]	2013	Case series	25	19:6	38.6	NA	12
Maffulli [56]	2012	Case series	21	16:5	47 (40–62)	20.6 (9.3–38.6)	130.8 (96–144)
Maffulli [57]	2014	Case series	28	21:7	Median 46	NA	24
Maffulli [58]	2010	Case series	32	28:4	47.1 (40–62)	16.3 (8.6–38.6)	24
Maffulli [59]	2013	Case series	26	23:3	42 (40–56)	16.3 (8.6–38.6)	98.4 (84–120)
Maffulli [60]	2012	Case series	16	16:0	55.6 (42–79)	20.9 (6.1–39.1)	186 (156–216)
Maffulli [61]	2017	Cohort	62	39:23	44.8 (29.3–62)	17.2 (8.6–35.6)	35.4 (25–49)
Maffulli [62]	2005	Case series	21	16:5	NA	20.9 (9.3–39.1)	28.4 ± 3.5
Mahajan [63]	2009	Case series	36 (38 feet)	24:12	70 (56–78)	15 (12–24)	12
Mann [64]	1991	Case series	7	4:3	33–66	13.0–156.4	39
Mao [65]	2015	Case series	10	8:2	35.5(22–55)	23.0 (17.4–34.8)	18.1 (12–36)
Miao [66]	2016	Case series	35	21:14	42.1 (23–71)	7.4 (4.1–146.4)	32.2 (18–72)
Miskulin [67]	2005	Case series	5	4:1	49.4	19.8 (6–40)	12
Mulier [68]	2003	Cohort	19	15:4	39.4	NA	18
Nambi [69]	2020	Case series	5	4:1	42.2 (11–72)	NA	3–12
Oksanen [70]	2014	Case series	7	4:3	53 (37–69)	68.6 (12.9–222.6)	27 (16–39)
Ozan [71]	2017	Case series	15	13:2	35.2 (22–42)	6 (4.3–8.6)	35.4 (25–49)
Ozer [72]	2018	Case series	19	18:1	47.4 (24–74)	5.8 (4–8.6)	8.3 (6.1–75)
Park [73]	2012	Case series	12	11:1	31–74	4.3–52.1	36.2 (13–94)
Parsons [74]	1989	Cohort	12	NA	43.2	200.6 (8.6–1300)	12
Pendse [75]	2019	Case series	16 (17 feet)	12:4	65.7 (51–82)	31.5 (13–65)	27 (17–52)
Pintore [76]	2001	Case series	22	21:1	41.3 (25–64)	20.9 (6.1–39.1)	53 (28–107)
Pavan Kumar [77]	2013	Case series	78	48:30	38–66	52.1 (13–156.4)	12
Rahm [78]	2013	Cohort	31 (32 feet)	18:14	54.1 (30–78)	NA	53.4 (13–135)
Sarzaeem [79]	2011	Case series	11	11:0	NA	52.1 (13–156.4)	25 (18–30)
Seker [80]	2016	Case series	21	NA	32.1 (17–45)	8.4 (4–48)	145.3 (121–181)
Shoaib [81]	2017	Case series	7	3:4	50.3 (36–66)	15 (6–24)	29.4 (24–36)
Song [82]	2020	Case series	34	30:4	36.1 (25–50)	NA	53 (24–80)
Takao [83]	2002	Case series	10	6:4	51 (38–57)	17.1 (8.6–30)	26–192
Tay [84]	2010	Case series	9	7:2	59.5 (54–75)	NA	24
Usuelli [85]	2017	Case series	8	5:3	50.5 (36–60)	5.8 (4.4–8.6)	27.9 (24–34)
Vega [86]	2018	Case series	22	16:6	69 (59–84)	NA	30.5 (18–46)
Wapner [87]	1994	Case series	7	NA	52	NA	17 (3–30)
Wegrzyn [88]	2010	Case series	11	7:4	44 (27–70)	NA	79
Winson [89]	2020	Case series	19	16:3	60 (39–80)	8.7 (2–35.6)	79.2 (49.2–111.6)
Yasuda [90]	2016	Case series	30	16:4	52.7 (17–78)	22 (5–70)	33 (24–43)
Yasuda [91]	2007	Case series	6	4:2	47 (17–60)	22 (9–30)	31 (24–43)
Yeoman [92]	2012	Case series	11	6:5	52.6 (30–70)	26.6 (6–104)	Minimum 6

NA, not available

## Results

**Fig. 1:**
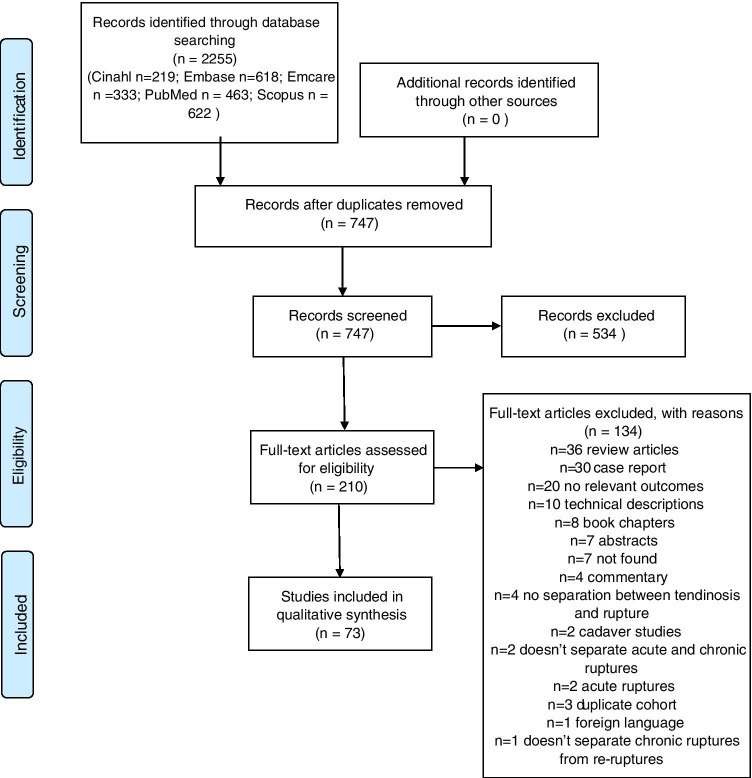
PRISMA flow diagram displaying the number of studies retrieved following searching and removed at each screening stage

A total of 747 unique articles were identified, of which 73 (10.3%) were included in the final review (Fig. 1). Summary statistics of all included studies are presented in Table 1.

### Treatment techniques

**Fig. 2:**
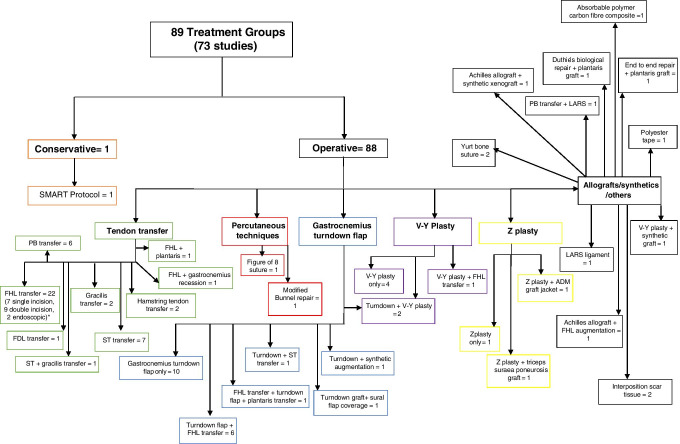
Flowchart detailing the number of studies using a particular treatment technique. FHL flexor hallucis longus, FDL: flexor digitorum longus: Semitendinosus, LARS: Ligament Advanced Reinforcement System (LARS) graft (JK Orthomedic, Dollard-des-Ormeaux, Quebec, Canada. *Two studies used both a single and a double incision FHL transfer approach in different patients, seven exclusively used a single incision, nine a double incision, two an endoscopic approach and two did not specify the exact approach

A wide variety of treatment methods were reported in the included literature, as detailed in Fig. 2. The most common technique is flexor hallucis longus (FHL) tendon transfer, reported in a total of 22 studies. Of these, two studies used both a single and a double incision approach in different patients, seven exclusively used a single incision, nine a double incision, two an endoscopic approach and two did not specify the exact approach. Other tendon transfer methods, such as semitendinosus tendon transfer (ST transfer), peroneus brevis tendon transfer (PB transfer) and hamstring tendon transfer, were reported in seven, six and two studies, respectively. Percutaneous techniques, including a figure of eight stitch repair or modified Bunnell repair, were reported in two studies [30, 52]. A total of ten studies used gastrocnemius flaps with no augmentation, whilst six studies describe additional FHL augmentation (Fig. 2). Techniques such as V–Y and Z plasty were reported both as stand-alone techniques or combined with a synthetic acellular human dermal tissue matrix graft jacket (Wright Medical Technology, Inc., Arlington, TN) or FHL transfer [53]. Other less commonly reported techniques include use of the Ligament Advanced Reinforcement System (LARS) graft (JK Orthomedic, Dollard-des-Ormeaux, Quebec, Canada), polyester tape, scar tissue interposition and Duthie’s biological repair [10, 43, 45, 91]. Only one study described nonoperative treatment, using an orthosis as part of the SMART protocol [89].

### Outcome measures

A similarly wide variety was seen in the outcome measures used to assess treatment outcomes. AOFAS is the most commonly used score, followed by ATRS, Leppilahti score and VAS (Table 2).

**Table 2:** Description of the outcome measures reported in included studies; some studies used more than one outcome measure to assess treatment results

Scale	Number of studies
Victorian Institute of Sports Assessment self-administered Achilles questionnaire (VISA-A)	3
Tegner activity scale	3
SF-36	6
Parson criteria	1
Mann criteria	3
Leppilahti score	9
Hooker scale	1
Foot Function Index (FFI)	1
Foot and Ankle Outcomes Instrument (FAOI) core/shoe comfort scale	1
Foot and Ankle Outcome Score (FOAS)	1
Foot and Ankle Ability Measure (FAAM) sports subscale	1
Boyden four-point scale	7
ATRS	21
AOFAS	43
(Visual Analogue Scale) VAS	9
(Foot and ankle Disability index) FADI	1
(Achilles Repair Score) ARS	1

### Treatment outcome

The outcomes of treatment using different techniques are detailed in Table 3. All 32 studies reporting both pre- and post-operative outcome measures found significant improvements in all measures used, with the exception of Koh et al., which found a significant improvement in AOFAS and VAS and SF-36 physical subscale but not SF-36 mental subscale score [50].

**Table 3:** Detailed breakdown of treatment techniques, associated outcomes and statistically significant changes between pre- and post-operative outcome scores

Author	Treatment	Mean pre-operative scores	Mean post-operative scores	Significant improvement?
Abubeih	FHL transfer	AOFAS: 57.4 ± 10.3	AOFAS: 95.3 ± 4.4	Yes P < 0.001
Ahmad	Central turndown + FHL transfer	FAAM: 36.3 (17–60) VAS: 6.6 (2–9)	FAAM: 90.2 (75–100) VAS: 1.8 (0–4)	Yes both P < 0.05
Alhaug	FHL transfer	NR	AOFAS: 87 (60–100) VISA-A: 81 (37–99)	NA
Arthur	FHL transfer	NR		
Badalihan	Yurt Bone suture	NR	Leppilahti: 100.0 ± 0.0	NA
Bai	Gastrocnemius turndown flap	NR	AOFAS: 92.6 ± 3.0 Leppilahti: 94.7 ± 3.1	
	Hamstring tendon transfer	NR	AOFAS: 93.5 ± 2.1 Leppilahti: 95.1 ± 3.1	No significant difference between the two treatment groups
Baumfield	Endoscopic FHL transfer	ATRS: 17.8 (11–28)	ATRS: 83.3 (79–87)	NR
Becher	End to end repair with plantaris tendon (10 patients), z plasty (2), turn down flap (1) or FHL transfer (1)	NR	ATRS: 75 ± 24 VISA-A: 81 ± 17 VAS: 0.8 ± 0.9	
Berteli	Percutaneous figure of 8 suture	NR	AOFAS: 99 (80–100)	NA
Borah	Gastrocnemius turndown flap		Leppilahti: 80–95	
Coull	FHL transfer	NR	AOFAS hallux metatarsophalangeal-interphalangeal: 97 (85–100) SF-36: 141.1 ± 3.98	NA
El Shazly	Endoscopic hamstring tendon graft	AOFAS: 32.6 ± 7.5	AOFAS: 90.8 ± 3.5	Yes P < 0.05
El-Shewy	2 gastrocnemius turndown flaps	AOFAS: 42.5 ± 2.4	AOFAS: 98.9 ± 3.6	Yes P = 0.003
Elgohary	FHL transfer + gastrocnemius recession	AOFAS: 65 (52–72)	AOFAS: 94 (76–100)	Yes P < 0.001
Elias	V–Y plasty + FHL transfer	AOFAS: 58.4 (34–77)	AOFAS: 94.1 (80–100)	Yes P < 0.001
Esenyel	Turndown flap + synthetic mesh	AOFAS: 64.8 ± 8.1	AOFAS: 97.8 ± 4.1	Yes P < 0.0001
Fotiadis	Duthie’s biological repair + plantaris transfer	NR	Leppilahti: 6 patients 90–100, remaining 3 scored 75–85	NA
Gedam	Turndown flap + ST augmentation	AOFAS: 64.5 (35–79) ATRS: 49.4 (30–70)	AOFAS: 96.9 (90–100) ATRS: 91.4 (83–97)	Yes P < 0.001 for both
Guclu	V–Y plasty + turndown flap	AOFAS: 63 ± 4	AOFAS: 95 ± 3	Yes P = 0.001
Hahn	FHL transfer	AOFAS: 60.3 (46–68)	AOFAS: 92 (71–100)	NR
Hollawell	Achilles allograft + synthetic xenograft	FAOI core: 53 ± 1 FAOI shoe comfort: 59 ± 0	FAOI core: 97 ± 1 FAOI shoe comfort: 10 ± 0	NR
Ibrahim 2007	PB transfer + LARS	AOFAS: NR Tegner: 2.7	AOFAS: 89 (54–100) Tegner:1.9	NR
Ibrahim 2009	LARS	AOFAS: 48.6 ± 12.7 Tegner: 2.58 ± 0.31	AOFAS: 85.9 ± 6.6 Tegner: 1.7 ± 0.29	Yes P = 0.001 both
Jain	Turndown flap + FHL transfer	AOFAS: 72.1 ± 8.3 ATRS: 61.7 ± 8.2	AOFAS: 98.4 ± 2.03 ATRS: 98 ± 1.9	Yes P = 0.001 both
Jennings	Polyester tape	Tegner: 2.7	Tegner: 1.8	Yes P < 0.05
Jiang	ST + gracilis graft	AOFAS: 54.3 (46–65) ATRS: 51.4 (40–61) SF-36 physical: 32.1 (25–35) SF-36 mental: 37.1 (32–40)	AOFAS: 97.6 (90–100) ATRS: 92.7 (83–100) SF-36 physical: 90 (80–95) SF-36 mental: 90.9 (84–96) VAS: 0	NR
Jielile	Yurt bone method + cast immobilisation		Leppilahti: 21/21 excellent at 2 years	NA
	Yurt bone method + active mobilisation		Leppilahti: 26/26 excellent at 2 years	NA
Khalid	FHL transfer	NR	AOFAS: 78.5 (54–94)	NA
Khiami	Z plasty + triceps surae aponeurosis graft	AOFAS: 63.6 ± 11.5	AOFAS: 96.1 ± 6.8	Yes P < 0.001
Koh	FHL transfer	AOFAS: 62 ± 22 VAS: 3 SF-36 physical: 39 ± 10 SF-36 mental: 55 ± 9	AOFAS: 90 ± 11 VAS: 0 SF-36 physical: 49 ± 9 SF-36 mental: 57 ± 12	Yes all P < 0.05 except SF-36 mental
	Turndown flap + FHL	AOFAS: 52 ± 19 VAS: 5 SF-36 physical: 33 ± 10 SF-36 mental: 51 ± 14	AOFAS: 95 ± 10 VAS: 0 SF-36 physical: 50 ± 9 SF-36 mental: 53 ± 17	Yes all P < 0.05 except SF-36 mental. No significant differences between treatment groups
Kosaka	PB transfer	NR	AOFAS: 86.9 ± 7.3	NA
Kosanovic	Percutaneous modified Bunnells’ repair	NA	Leppilahti: 83.3 (60–100) Excellent (11) Good (2), Fair (5),	NA
Lee	Z plasty + ADM graft jacket	AOFAS: 46.3 (27–64)	AOFAS: 86.2 (78–95)	Yes P < 0.001
Lin 2019	V–Y plasty	AOFAS: 59.3 ± 12.3 ATRS: 39.6 ± 14.2	AOFAS: 96.6 ± 3.8 ATRS: 94.1 ± 4.9	Yes P < 0.05 both
Lin 2016	V–Y plasty with turndown flap in some. FHL transfer in those with no stump integrity	AOFAS: 60.1 ± 10.6 ATRS: 43.8 ± 11.8	AOFAS: 94.6 ± 4.0 ATRS: 92.6 ± 7.8	Yes P < 0.05 both
Lins	ST tendon graft	NR	AOFAS: 85.2 ± 18.0	NA
Maffulli 2005	Gracilis tendon graft	NA	Boyden: Excellent (2), Good (15), Fair (4), Poor (0)	NA
Maffulli 2010	PB transfer	NR	ATRS: 92.5 ± 14.2 Boyden: Excellent (6), Good (24), Fair (2)	NA
Maffulli 2013	ST transfer	NR	ATRS: 88 (75–97) Boyden: Excellent (10), Good (13), Fair (3)	NA
Maffulli 2012	PB transfer	NR	ATRS: 89.5 ± 12.2 Boyden: Excellent (4), Good (9), Fair (3)	NA
Maffulli 2017	ST transfer	ATRS: 50.4 ± 7.5	ATRS: 89.4 ± 3.2	Yes P < 0.001
	PB transfer	ATRS: 51.3 ± 4.5	ATRS: 89.5 ± 4.1	Yes P < 0.001
	FHL transfer	ATRS: 52.3 ± 3.2	ATRS: 88.9 ± 3.1	Yes P < 0.01, no significant difference between treatment groups
Maffulli 2012	Gracilis graft	NR	ATRS: 90.1 ± 5.8 Boyden: Excellent (2), Good (15), Fair (4),	NA
Maffulli 2014	ST graft + interference screw fixation	ATRS: 42(29–55)	ATRS: 86 (78–95) Boyden: Excellent (5), Good (21), Fair (2)	Yes P < 0.001
Mahajan	FHL transfer	AOFAS: 69 (58–76)	AOFAS: 88 (79–94)	Yes P < 0.001
Mann	FDL transfer	NA	Mann criteria: Excellent (4), Good (2), Fair (1)	NA
Mao	FHL transfer + 2 turndown flaps + plantaris augmentation	AOFAS: 64.4 ± 3.5 VAS: 4.33 ± 1.1	AOFAS: 94.3 ± 3.5 VAS: 1.89 ± 1.2	AOFAS: P = 0.008 VAS: P = 0.011
Miao	FHL transfer	AOFAS: 51.9 ± 7.1 Leppilahti: 72.6 ± 7.43	AOFAS: 92.6 ± 6.7 Leppilahti: 92.66 ± 5.1	Yes P < 0.05 both
Miskulin	PB transfer	NA	Mann criteria: Excellent (5)	NA
Mulier	Turndown flap	NA	Leppilahti: 62 (48–78)	NR
	Turndown flap + FHL transfer	NA	Leppilahti: 77 (67–89)	NR
Nambi	Turndown flap + sural flap	NR	ATRS: 70 (65–76)	NA
Oksanen	FHL transfer	NR	ATRS: 70 (38–96)	NA
Ozan	Turn down flap or V–Y plasty	NR	Hooker scale: Excellent (11), Satisfactory (4)	NA
Ozer	FHL transfer	NR	93.8	NA
Park	V–Y plasty ( 1), turndown flap (3), FHL transfer (3), allograft + FHL transfer (2)	AOFAS: 68.7 (50–87) VAS: 6.5 (5–8)	AOFAS: 98 (88–100) ATRS: 92.9 (84–100) VAS: 0.2	Yes P < 0.001 both
Parsons	Polymer carbon fibre composite	Parson’s score: 24.5	Parson’s score: 45.5	Yes P < 0.05
Pavan Kumar	Turndown flap	NR	Leppilahti: Excellent (62). Good (8), fair (4), poor (2)	NA
Pendse	FHL transfer	AOFAS: 57.5 ± 6.0	AOFAS: 96.7 ± 3.6	Yes P < 0.001
Pintore	PB transfer	NR	Boyden: Excellent (15), Good (3), Fair (4)	NA
Rahm	FHL transfer	AOFAS: 62.4 (32–87)	AOFAS: 86.9 (43–100) SF-36: 71.7% (28%-95%) VISA-A: 70.3 (20–97) FFI pain:20.2% (0–81%) FFI function: 23.0% (0–70%)	Yes P < 0.001
Sarzaeem	ST transfer	AOFAS: 70 ± 5 ATRS: 32 ± 6	AOFAS: 92 ± 5 ATRS: 89 ± 4	Yes P = 0.001 both
Seker	Turndown flap	NR	AOFAS: 98.5(90–100) FADI: 98.9% (96.2–100%) VAS: 0	NA
Shoaib	V–Y plasty + Artelon synthetic graft	AOFAS: 59.4 (31–73)	AOFAS: 91.5 (67–100) ATRS: 92.1 (79–100) VAS pain: 0 in all VAS function: 8 (7–9)	AOFAS: Yes P = 0.018
Song	ST transfer	AOFAS: Median 50 (5–75)	AOFAS: Median 100 (86–100)	Yes P < 0.05
Takao	Turndown flap	AOFAS: 72.6 ± 5.3	AOFAS: 98.1 ± 2.5	Yes P < 0.0001
Tay	Two turndown flaps + FHL transfer	NR	AOFAS: 94.2 (78–100) SF-36 physical: 88.3 SF-36 mental: 90.7 Vas: 0.8 (0–5)	NA
Usuelli	ST transfer	NR	AOFAS: 92 (83–96) ATRS: 87 (81–95)	NA
Vega	Endoscopic FHL transfer	AOFAS: 55 (26–75)	AOFAS: 91(74–100)	NR
Wapner	FHL transfer, 2 patients received plantaris augmentation and 1 a turndown flap	NR	Mann criteria: Excellent (3), good (3), fair (1)	NA
Wegrzyn	FHL transfer	AOFAS: 64 (58–80)	AOFAS: 98 (90–100)	Yes P < 0.001
Winson	SMART conservative	NR	ATRS:83 (39–100) ARS: 77.5 (35–100)	NA
Yasuda 2016	Scar tissue interposition	AOFAS: 82.8 ± 8.3	AOFAS: 98.1 ± 3.9 ATRS: 92 (80–100)	NR
Yasuda 2007	Scar tissue interposition	AOFAS: 88.2	AOFAS: 98.3	Yes P = 0.0277
Yeoman	FHL transfer	AOFAS: 51.4 (26–87) SF-36: 87.4 (75.4–109.5)	AOFAS: 91.9 (77–100) SF-36: 111.8 (103.9–116.2)	NR

Only two treatment techniques met the outlined pooling criteria. A total of eight studies describing FHL transfer showed a mean pre-operative AOFAS of 62.3 (95% CI: 57.1–67.4) and mean post-operative AOFAS of 94.2 (95% CI: 90.9–97.4), giving an unstandardised mean difference of 31.9 [22, 50, 63, 66, 75, 78, 88, 92]. Unfortunately, there were an insufficient number of studies reporting the same treatment outcome to specifically compare outcomes seen using a single or double incision approach. Three studies describing semitendinosus transfer show a pooled mean ATRS of 40.8 (95% CI: 30.4–51.1), post-operative ATRS of 88.5 (95% CI: 84.2–92.9) and mean difference of 47.7 [57, 61, 79]. No formal comparison of these mean differences was performed, due to the heterogeneity in outcome measure and low-level case series study design used and inability to control for potential confounding factors such as treatment delay, length of tendon gap, patient age and follow-up period.

### Complications

A total of 50 studies involving 1063 patients (1065 feet) clearly reported treatment complications (Table 4). Complications were categorised as infection (superficial wound infection, deep infection), wound healing (wound dehiscence, delayed wound closure, hypertrophic scar, wound breakdown, wound gaping), tendon re-rupture and others. The overall pooled complication rate was 168/1065 (15.8%), with the most common complication being infection (58/1065, 5.5%).

**Table 4:** Detailed breakdown of post-intervention complications reported in included studies

Author	Total patients	Infection		Wound healing	Re-rupture	Other	Total
Abubeih	21		1 (4.7%)	0	0	0	1/21 (4.7%)
Ahmad	32		1 (3.1%)	3 (9.4%)	0	1 (3.1%) DVT	5/32 (15.6%)
Alhaug	21		5 (23.8%)	2 (9.5%)	1 (4.8%)	2 (9.5%) clawed toes, 6 (28.6%) hypoesthesia	16/21 (76.2%)
Arthur	7		2 (28.6%)	1 (14.3%)	0	0	3/7 (42.9%)
Badalihan	51		0	0	0	5 (9.8%) local ischaemic necrosis	5/51 (9.8%)
Bai	26		2 (7.7%)	0	0	1 DVT (3.8%), 1 (3.8%) saphenous nerve injury	4/26 (15.4%)
Borah	5		1 (20%)	0	0	0	1/5 (20%)
El shewy	11		2 (18.2%)	3 (27.3%)	0	0	5/11 (45.5%)
Elias	15		0	1	0	0	1/15 (6.7%)
Esenzyel	10		0	0	0	0	0%
Gedam	14		0	0	0	0	0%
Guclu	17		2 (11.7%)	0	0	0	2/17 (11.7%)
Hollawell	4		0	0	0	0	0%
Ibrahim 2007	13		0	0	0	2 (15.4%) skin necrosis	2/13 (15.4%)
Ibrahim 2009	14		1 (7.1%)	0	0	0	1/14 (7.1%)
Jain	15		1 (6.7%)	0	0	1 stiffness (6.7%), 2 (13.3%) sterile serous discharge	4/15 (26.7%)
Jennings	16		3 (18.8%)	0	0	1 (6.3%) hypoesthesia	4/16 (25%)
Jielile	57		4 (7.0%)	0	6 (10.5%)	6 (10.5%) calcified tendon plaques, 2 (3.51%) ischaemic necrosis	18/57 (31.6%)
Khalid	10		1 (10%)	0	0	0	1/10 (10%)
Khiami	23		0	2 (8.7%)	0	1 (4.3%) hypoesthesia	3/23 (13.0%)
Koh	49		1 (2.0%)	1 (2.0%)	0	0	2/49 (4.1%)
Kosaka	20		0	0	0	0	0%
Kosanovic	22		0	0	0	1 DVT (4.5%), 1 (4.5%) poor tendon healing	2/22 (9.1%)
Lee	9		0	3 (33.3%)	0	1 (11.1%) DVT	4/11 (44.4%)
Lin 2016	29		0	0	0	0	0%
Lin 2019	20		0	0	0	0	0%
Maffulli 2005	21		5 (23.8%)	1 (4.8%)	0	2 (9.5%) hypersensitivity	8/21 (38.1%)
Maffulli 2010	32		4 (12.5%)	2 (6.3%)	0	3 (9.4%) toe clawing	9/32 (28.1%)
Maffulli 2012	21		3 (14.3%)	1 (4.8%)	0	1 (4.8%) tendinopathy	5/21 (23.8%)
Maffulli 2013	26		1 (3.8%)	0	0	1 (3.8%) tendinopathy, 2 (7.7%) hypersensitivity	4/26 (15.4%)
Maffulli 2014	28		0	0	0	0	0%
Mahajan	36 (38 feet)		3 (7.9%)	1 (2.6%)	0	3 (7.9%) weak push off	7/38 (18.4%)
Mao	10		0	0	0	0	0%
Mulier	19		1 (5.3%)	0	1 (5.3%)	2 (10.5%) DVT, 2 (10.5%) hypoesthesia	6/19 (31.6%)
Nambi	5		0	0	0	5 (100%) hypoesthesia	5/5 (100%)
Ozan	15		0	0	0	0	0%
Park	12		0	0	0	0	0%
Parsons	52		5 (9.6%)	0	0	1 (1.9%) tendonitis	6/52(11.5%)
Pavan Kumar	78		3 (3.85%)	5 (6.4%)	0	0	8/78 (10.3%)
Rahm	31		1 (3.2%)	5 (16.1%)	1 (3.2%)	1 (3.2%) DVT, 1 (3.2%) suture granuloma	9/31 (29.0%)
Sarzaeem	11		2 (22.2%)	0	0	1 (11.1%) DVT	3/11 (33.3%)
Seker	21		1 (4.8%)	0	0	0	1/21 (4.8%)
Shoaib	7		1 (14.3%)	0	0	3 (42.9%) hypoesthesia	4/7 (57,1%)
Takao	10		0	0	0	0	0%
Tay	9		0	0	0	1 (11.1%) neuropraxia, 2 (22.2%) hypoesthesia	3/9 (33.3%)
Vega	22		0	0	0	1 (4.5%) calcaneal fragment avulsion	1/22 (4.5%)
Winson	19		0	0	0	1 (5.3%) PE	1/19 (5.3%)
Yasuda 2007	6		0	1 (16.7%)	0	0	1/6 (16.7%)
Yasuda 2016	30		0	1	0	0	1/30 (3.3%)
Yeoman	11		1 (9.1%)	0	0	1 (9.1%) DVT	2/11 (18.2%)
Total	1063 (1065 feet)		58 (5.45%)	33 (3.10%)	9 (0.85%)	68 (6.38%)	168 (15.8%)

## Discussion

The aim of this scoping review was to systematically map and summarise current literature describing the treatment of chronic Achilles tendon ruptures. A previous systematic review on the same subject, performed in 2012 by Hadi et al., included 34 studies [12]. Since then, there appears to have been a surge in publications on the topic, with 43 of the 73 (58.9%) included in this review published in 2013 onwards (Table 1). Unfortunately, despite this surge in the number of publications, the quality and level of evidence has not risen. As in the review of Hadi et al., the majority of included studies are level IV evidence case series, with only seven comparative cohort studies identified [12].

There is a large degree of heterogeneity in treatment methods for chronic Achilles ruptures, with studies reporting a variety of tendon transfer, turndown flap, tendon lengthening and synthetic repair techniques. A number of authors also described the use of dual techniques involving a combination of more than one of the above methods. All techniques described appeared to show good post-operative results, with all relevant included studies reporting a statistically significant increase in pre- to post-operative scores such as AOFAS and ATRS (Table 3). However, ascertaining the most efficacious technique is challenging, due to the poor quality of the existing literature. A formal meta-analysis comparing pooled outcomes of different treatment strategies was not possible due to a number of factors including large number of different techniques, large variety in outcome measures reported, low-level case series study design and inability to control for factors which may influence outcomes such as patient age, length of treatment delay and length of tendon gap. Comparison is also currently hampered by the widespread use of non-validated outcome measures. The most commonly used measure was the AOFAS (Table 2), which is not validated for use in Achilles ruptures and its use is no longer recommended by The American Orthopaedic Foot and Ankle Society [93]. Future research should therefore endeavour to use outcome measures specifically validated for Achilles ruptures such as the ATRS.

However, even if such a comparison between treatment techniques was possible, it is likely that there is no a single optimal operative strategy for all patients. Instead, it may be more important to develop an evidence-based optimal treatment protocol, identifying stratification criteria that takes into account unique patient factors, such as length of treatment delay and tendon gap size, which may determine the suitability of a particular technique. Some authors have described such treatment protocols. For example, Myerson recommends primary repair in cases with < 2 cm gap, V–Y plasty in the case of a 2–5 cm gap and tendon transfer with or without V–Y plasty in cases with gap > 5 cm [94]. Maffulli et al. use peroneus brevis transfer for gaps < 6 cm, semitendinosus graft for gaps > 6 cm and FHL transfer for gaps > 5 cm [61]. Similar gap size-based protocols are also described by Kuwada, Den Hartog and Krahe [95–97]. However, these protocols are not based on definitive evidence as there is currently a lack of literature comparing different treatment methods. Although Elias et al., who described FHL transfer, did not find any significant difference in outcomes according to age or length of delay, worse outcomes were seen in those with larger tendon gaps of 7–8 cm [97]. However, firm conclusions cannot be drawn from the findings of these 15-patient case series describing only one technique. It is therefore important that further high-quality research, comparing different treatment techniques in patients of varying age, tendon gap length, treatment delay, injury aetiology and degree of tendon degeneration, is performed. Such works would aid the development of an evidence-based treatment protocol, which would allow clinicians to select the optimal technique for each specific patient, taking into account the above factors.

Furthermore, although there is a growing body of evidence supporting the role of conservative treatment in acute Achilles rupture, there is a paucity of literature investigating the same in chronic ruptures [9]. This is likely due to the traditional view that operative treatment yields superior outcomes for chronic ruptures. However, again, this seems to be derived from anectodical evidence rather than high-quality research. Only one included study investigates the role of conservative treatment and, to the best of our knowledge, the only article directly comparing operative versus conservative treatment in chronic ruptures is the 1953 study of Christensen [89, 98]. This study does indeed suggest superiority of operative treatment; however, it is not possible to draw conclusions from a single small case series. Further research is therefore required in ascertaining the suitability of conservative treatment and specific factors which may predict response to such treatment. Even if it is the case that operative treatment is superior, there may be certain patients who decline, or are not suitable for operative intervention. Although Achilles tendon rupture most frequently occurs in adults aged between 30 and 40, there are a group of older patients sustaining Achilles tendon rupture who may not be able to tolerate surgery and the mean age at which rupture occurs has increased by at least 0.721 years every five years since 1953 [99]. This suggests that clinicians are likely to come across an increasing number of patients for whom operative intervention is not suitable, further emphasising the importance of research into the development of effective conservative therapies.

Despite the rigorous methodology employed in this review, it must be acknowledged that certain biases do exist. For example, due to the limited linguistic capabilities of the research team, only studies published in the English language were included. Furthermore, as described, there are a number of confounding factors such as treatment delay and tendon gap length, which may differ between individual studies and affect reported outcomes. As outlined in Table 1, the large majority of studies utilise a level IV retrospective case series design. Such studies are particularly prone to selection bias, drawing patients from a relatively narrow sample population. Lastly, it was decided to include studies reporting both validated and non-validated outcome scores, as well as patient reported, and researcher assessed scores. This may cause some bias in outcome scores, with only 21 of 73 included studies using the validated ATRS outcome scale.

## Conclusion

The current literature describes a number of different operative strategies for the management of chronic Achilles rupture, all of which demonstrate beneficial outcomes. However, comparison of specific techniques is currently hampered by the low-level evidence and inability to control for potential confounding factors. Future research directly comparing treatment strategies in patients stratified according to specific injury characteristics may aid in the development of an evidence-based optimal treatment protocol. This would allow clinicians to determine which of the multitude of available techniques is most suitable for each unique patient.

## Supplementary information

ESM 1 (DOCX 20 kb)

## Data Availability

All data is freely available online.
