# Supplementary material for: Management of chronic Achilles ruptures: a scoping review
Source: Int Orthop. 2021 Jun 5;45(10):2543–59. doi: 10.1007/s00264-021-05102-5 (PMC8514369; doi:10.1007/s00264-021-05102-5)
Supplement: Supplementary file 1 — (DOCX 20 kb) [file 264_2021_5102_MOESM1_ESM.docx]

| Search Terms | Number of Results |
| --- | --- |
| 1. (Chronic OR neglected) AND (Achilles OR tendoachill* OR calcaneal tendon) AND (rupture) | 463 |
| 1. Achilles tendon [MeSH] AND (chronic OR neglected) AND (rupture) | 363 |
| 1. 1 OR 2 | 463 |

Search Strategy

Online Resource 1: Table detailing the search strategy used and number of search results. Number of results shown is for PubMed only, with an English language filter applied.
